# An Amperometric Biomedical Sensor for the Determination of Homocysteine Using Gold Nanoparticles and Acetylene Black-Dihexadecyl Phosphate-Modified Glassy Carbon Electrode

**DOI:** 10.3390/mi14010198

**Published:** 2023-01-13

**Authors:** Chunnan Zhu, Jingfang Zhang, Shunrun Zhang, Chao Liu, Xiaojun Liu, Jian Jin, Dongyun Zheng

**Affiliations:** 1 College of Biomedical Engineering, South-Central Minzu University, Wuhan 430074, China; 2 Key Laboratory of Brain Cognitive Science (State Ethnic Affairs Commission), South-Central Minzu University, Wuhan 430074, China; 3 Hubei Key Laboratory of Medical Information Analysis and Tumor Diagnosis & Treatment, Wuhan 430074, China; 4 The First Hospital of Wuhan, Wuhan 430022, China

**Keywords:** gold nanoparticles, acetylene black, dihexadecyl phosphate, glassy carbon electrode, homocysteine, detection

## Abstract

A novel nanocomposite film composed of gold nanoparticles and acetylene black–dihexadecyl phosphate was fabricated and modified on the surface of a glassy carbon electrode through a simple and controllable dropping and electropolymerization method. The nanocomposite film electrode showed a good electrocatalytic response to the oxidation of homocysteine and can work as an amperometric biomedical sensor for homocysteine. With the aid of scanning electron microscopy, energy dispersive X-ray technology and electrochemical impedance spectroscopy, the sensing interface was characterized, and the sensing mechanism was discussed. Under optimal conditions, the oxidation peak current of homocysteine was linearly increased with its concentration in the range of 3.0 µmol/L~1.0 mmol/L, and a sensitivity of 18 nA/(μmol/L) was obtained. Furthermore, the detection limit was determined as 0.6 µmol/L, and the response time was detected as 3 s. Applying the nanocomposite film electrode for monitoring the homocysteine in human blood serum, the results were satisfactory.

## 1. Introduction

The abnormal elevation of the homocysteine (HcySH) concentration in the blood characterizes hyperhomocysteinemia [1], which leads to coronary heart disease [2], cardiovascular and cerebrovascular disease [3], kidney disease [4], sarcopenia [5] and mental illness [6]. Therefore, the accurate and rapid measurement of homocysteine is important for the prevention and diagnosis of related diseases. Some techniques, including high performance liquid chromatography [7], chromatography–mass spectrometry [8] and capillary electrophoresis [9] have been applied for the detection of HcySH with good specificity and a low detection limit. However, expensive instrumentation and time-consuming sample preparation are needed for these techniques. By comparison, electrochemical sensing methods are a superior option due to their simple operation, low cost, high sensitivity, rapid response and real-time analysis [10].

Sensitive functional materials play a significant role in ensuring the performance of electrochemical sensors [11]. One of the most applied, sensitive, functional materials is a nanomaterial that has a large specific surface area and unique photoelectric properties [12]. Some electrochemical sensors have been developed using nanomaterials and then have been applied for measuring HcySH with good results [13]. However, some performance factors, including sensitivity, selectivity and response time, still need improvement to meet the requirements of clinical laboratory medicine. Therefore, constructing a novel HcySH nanosensing interface with better performance is essential for a biomedical HcySH sensor.

As a special type of carbon black with a regular porous structure, acetylene black (AB) has excellent electrical conductivity, a large surface area and a strong adsorption capacity [14]. In addition, compared with other carbon materials, such as carbon nanotubes, graphene and carbon nanofibers, etc., the ultra-low cost of AB helps to maintain the low cost of composite materials [15], which makes its widespread application in electrochemical sensing fields possible [16,17,18,19].

Gold nanoparticles (AuNPs), important metal nanomaterials, are useful for roughening the sensing interface, catalysis, electrical conductivity and enhanced mass transfer [20]. Therefore, it is widely used in the preparation of chemically modified electrodes. AuNPs are obtained by chemical, physical and biosynthetic methods [21,22]. Among them, the electrodeposition method provides a simple, fast and inexpensive alternative. In order to improve the utilization rate of AuNPs and enhance their catalytic activity, AuNPs are usually loaded onto high-conductivity carbon nanomaterials to expose more of their active catalytic sites during electrochemical sensing and, thereby, increasing their sensitivity and the stability of the sensor [23].

Dihexadecyl phosphate (C_32_H_67_O_4_P, DHP) is an anionic hydrophobic surfactant. It has two long hydrocarbon chains bonded with negatively charged phosphate esters, which self-assemble into multiple bilayer structures, similar to lipid bilayers. Due to the existence of hydrophobic interaction, it forms a stable coating on the electrode surface. This behavior allows for the dispersion of nanomaterials (carbon nanotubes, acetylene black, etc.) and the preparation of stable aqueous solutions, and it has been used to prepare homogeneous suspensions [24,25,26].

In 2018, Rajaram and Mathiyarasu reported an electrochemical sensor for the quantification of HcySH using Au nanoparticles incorporated into a reduced graphene oxide -modified glassy carbon electrode (AuNP/rGO/GCE) [27]. A significant advantage of the reported sensor was the low overpotential that was beneficial for good selectivity. However, graphene is expensive, and the preparation of graphene oxide is complex. Moreover, the linear range of the reported sensor was from 2 mM to 14 mM, which does not cover the normal level of blood HcySH (5~16 µM) and, thus, restricted its biomedical application. Therefore, our group aimed to develop an electrochemical sensor for the detection of blood HcySH with a better potential for biomedical applications. In this work, the low-cost carbon nanomaterial acetylene black was used for the first time for the electrochemical sensing of homocysteine. After fabricating a AuNPs/AB–DHP/GCE, the electrocatalytic oxidation of HcySH on the surface of the AuNPs/AB–DHP was studied. A new type of HcySH electrochemical nanosensor was constructed. The results of the SEM and electrochemical experiments showed that the AuNPs/AB–DHP nanocomposite film had a loose and porous nanostructure on the surface of the GCE, which had a significant catalytic effect for the electrochemical oxidation of HcySH. The constructed HcySH biomedical sensor had good performance and could be used for the accurate determination of HcySH levels in human serum samples.

## 2. Materials and Methods

### 2.1. Chemicals and Apparatus

DL-HcySH (purity > 90%) was purchased from TCI Development Co., Ltd. (Shanghai, China). HAuCl_4_·4H_2_O was obtained from Shanghai Chemical Reagent Co., Ltd. (Shanghai, China). Acetylene black (AB, particle size 30~45 nm) was purchased from Tianjin Youmeng Chemical Technology Co., Ltd. (Tianjin, China). Dihexadecyl phosphate (DHP, C_32_H_67_O_4_P, purity > 90%) was obtained from Shanghai Aladdin Biochemical Technology Co., Ltd. (Shanghai, China). KCl (purity ≥ 99.5%) and KH_2_PO_4_ (purity ≥ 99.0%) were purchased from Tianjin Beilian Fine Chemicals Development Co., Ltd. (Tianjin, China). NaCl, (purity ≥ 99.5%), MgCl_2_·6H_2_O (purity ≥ 98.0%), CaCl_2_ (purity ≥ 96.0%) and Na_2_HPO_4_·12H_2_O (purity ≥ 99.0%) were obtained from Sinopharm Chemical Reagent Co., Ltd. (Shanghai, China). Dopamine (DA), ascorbic acid (AA) and uric acid (UA) were obtained from Sigma Aldrich (Shanghai) Trading Co., Ltd. (Shanghai, China) Normal human serum for scientific research was purchased from Shanghai Xinfan Biotechnology Co., Ltd. (Shanghai, China). All reagents were of analytical grade and used without further purification. All solutions were prepared with ultrapure water.

Preparation of AB–DHP dispersion: Adding 4 mg AB and 4 mg DHP into 4 mL ultrapure water and sonicating for 10 h to prepare 1.0 mg/mL AB–DHP aqueous dispersion.

CHI660D electrochemical workstation (Shanghai Chenhua Instrument Co., Ltd., Shanghai, China) was used for electrochemical experiments. A pHS-3E acidity meter (Shanghai Youke Instrument Co., Ltd., Shanghai, China) was used to adjust the pH value of the supporting electrolyte. Scanning electron microscopy (SEM) and energy dispersive X-ray spectroscopy (EDX) characterization were performed using an SU8010 field emission scanning electron microscope.

### 2.2. Preparation of Different Electrodes

**Figure 1:**
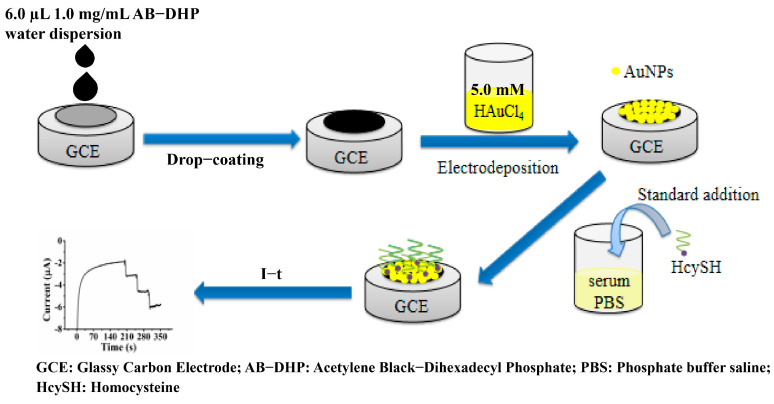
Diagram of the preparation process and application of the HcySH biomedical sensor.

Preparation of AuNPs/AB–DHP/GCE: Firstly, a bare glassy carbon electrode (GCE) was polished on 50 nm Al_2_O_3_ slurry and sonicated in 8.0 mmol/L (mM) HNO_3_ aqueous solution, ethanol and ultrapure water for 2 min, separately. Then, 6.0 µL 1.0 mg/mL AB–DHP aqueous dispersion was dropped on the surface of GCE and dried under infrared light to fabricate AB–DHP/GCE. Finally, Au nanoparticles were modified on the surface of AB–DHP/GCE through cyclic voltametric scanning of AB–DHP/GCE in 5.0 mM HAuCl_4_ aqueous solution from 0 V to 1.5 V at a scan rate of 100 mV/s for 25 cycles. The AuNPs/AB–DHP/GCE was obtained for use as a HcySH electrochemical sensor. The schematic diagram of the sensor preparation process is shown in Figure 1.

For comparison, AuNPs/GCE was prepared via a similar process, as described above, by replacing AB–DHP/GCE with bare GCE.

### 2.3. Electrochemical Detection of HcySH

Electrochemical detection of HcySH was carried out using a CHI660D electrochemical workstation with a conventional three-electrode system composed of bare GCE, AB–DHP/GCE, AuNPs/GCE or AuNPs/AB–DHP/GCE as the working electrodes; a saturated calomel electrode (SCE) as the reference electrode; and a platinum electrode as the counter electrode. The supporting electrolyte was 0.15 M phosphate buffer saline (PBS, pH = 6.0). The applied potential range was 0 V~0.7 V. The AuNPs/AB–DHP/GCE was washed with 0.15 M PBS (pH = 6.0) before re-use.

## 3. Results and Discussion

### 3.1. Electrochemical Response of HcySH on Different Electrodes

**Figure 2:**
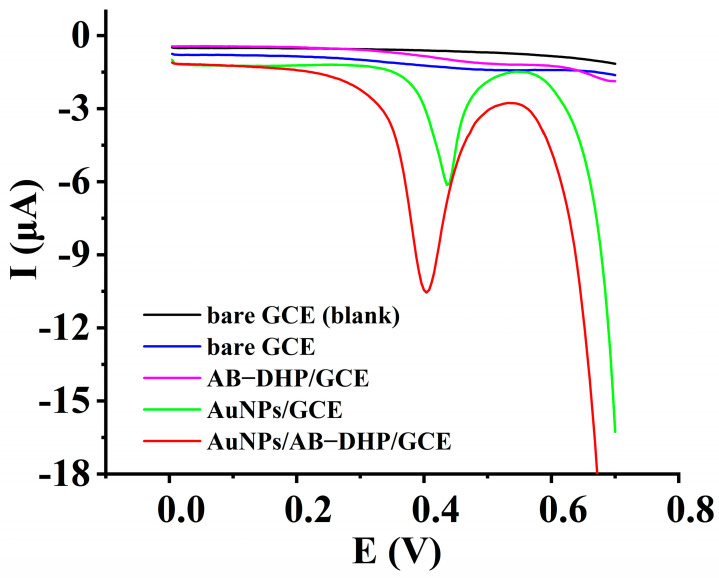
Differential pulse voltammograms between 0 μM and 200 μM HcySH on different electrodes in 0.15 M PBS (pH = 6.0).

To ensure the electrocatalytic response of the AuNPs/AB–DHP nanocomposite film to the oxidation of HcySH, the electrochemical behavior of HcySH on different electrodes was collected through differential pulse voltammetry, which is shown in Figure 2. When there was no HcySH in the supporting electrolyte, no electrochemical response appeared (black line). When there was 200 µM HcySH in the supporting electrolyte, no obvious electrochemical response was observed on the bare GCE (blue line), and only a weak oxidation peak (E_p_ = 0.51 V, I_p_ = 0.17 µA) was observed on AB–DHP/GCE (purple line). On AuNPs/GCE (green line), a sharp oxidation peak (E_p_ = 0.46 V, I_p_ = 4.7 µA) appeared, and a better oxidation peak (E_p_ = 0.4 V, I_p_ = 8.3 µA) was obtained on AuNPs/AB–DHP/GCE (red line). The results indicated that the AuNPs/AB–DHP/GCE showed an obvious electrocatalytic response to the oxidation of HcySH, which may be attributed to the large specific surface area of the AB, the good conductivity of the AuNPs and the affinity of the AuNPs to HcySH through the Au-S bond. 

### 3.2. Characterizations of Nanocomposite Film

#### 3.2.1. Characterization of Surface Topography

**Figure 3:**
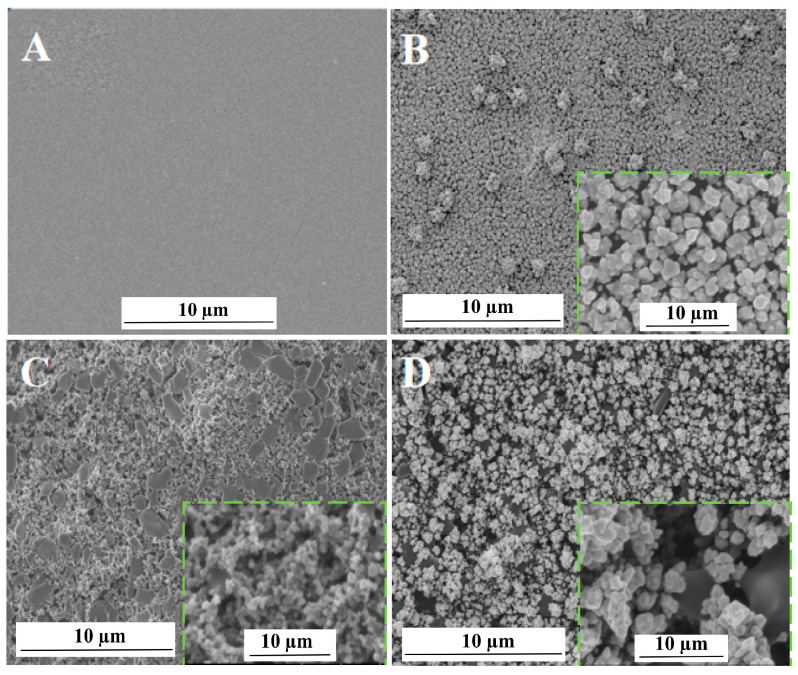
SEM images of bare GCE (**A**), AuNPs/GCE (**B**), AB–DHP/GCE (**C**) and AuNPs/AB–DHP/GCE (**D**).

The surface topography of different electrodes was characterized with the aid of scanning electron microscopy (SEM), and the results are shown in Figure 3. As seen in Figure 3A, the surface of the bare GCE was clear and smooth. On the surface of AuNPs/GCE, a large quantity of AuNPs was observed (Figure 3B). The AB–DHP film presented a compact block structure on the surface of the AB–DHP/GCE (Figure 3C). After electrodepositing the AuNPs on the surface of the AB–DHP/GCE, the entire electrode surface loosened (Figure 3D), and many AuNPs were attached on the surface of the porous AB. The nano-effect of the AuNPs, together with the relaxed and porous structure of the AB, increased the specific surface area of the electrode effectively, which was conducive to the enrichment of HcySH and, thereby, significantly improved the detection sensitivity of the sensor.

#### 3.2.2. Energy Dispersive X-ray Spectrum Analysis

**Figure 4:**
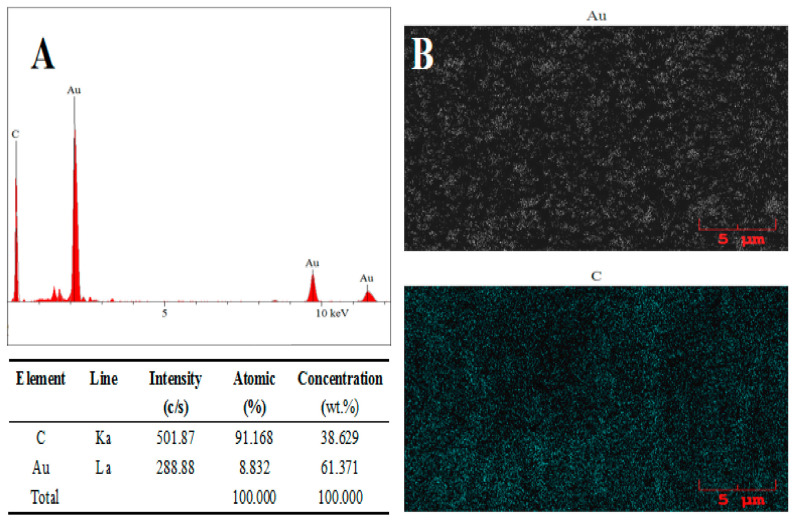
(**A**) EDX spectrum of AuNPs/AB–DHP/GCE. (**B**) EDX spectral mapping of the carbon (white) and gold (blue) at the same location.

Energy dispersive X-ray spectrum analysis was used to further confirm the successful addition of the AuNPs and the AB–DHP nanocomposite film, and the result is shown in Figure 4. Obviously, the elements present on the surface of the AuNPs/AB–DHP/GCE were C and Au (Figure 4A). The main source of the C was the AB and the glassy carbon. The Au was mainly derived from the AuNPs. Figure 4B shows the distribution map of the Au and the C on the surface of the electrode. In the figure, the white represents the Au, and the blue represents the C. It can be seen that the Au and the C were uniformly and widely distributed on the electrode surface, which showed that the AuNPs/AB–DHP composite film was successfully added to the surface of the GCE.

#### 3.2.3. Characterization of Electrochemical Impedance Spectrum

**Figure 5:**
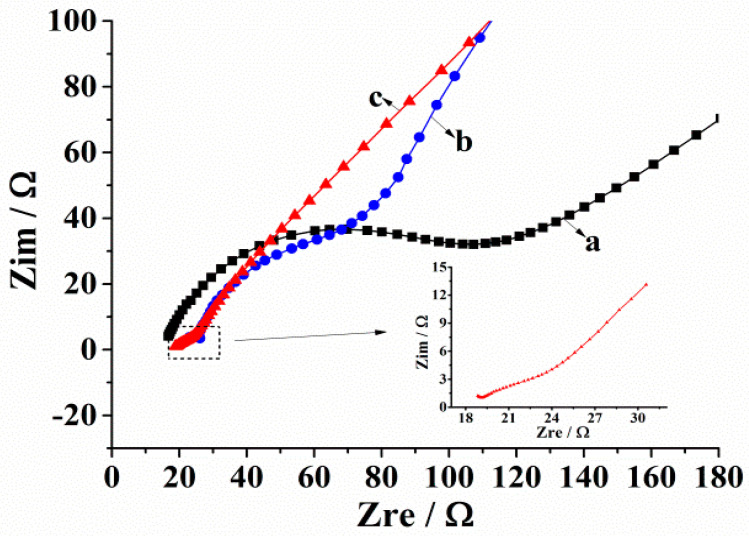
Electrochemical impedance spectroscopy of bare GCE (a), AB–DHP/GCE (b) and AuNPs/AB–DHP/GCE (c) in 5 mM Fe(CN)_6_^3−/4−^ and 1 M KCl solution.

With the aid of the electrochemical impedance spectrum (EIS), the impedance state of the electrode surface was characterized, and the corresponding Nyquist diagram is shown in Figure 5. A typical impedance spectrum includes a semicircular part at a higher frequency and a linear part at a lower frequency. The former corresponds to the charge transfer process on the electrode interface; moreover, the diameter of the semicircle is proportional to the impedance of the electron transfer during the electrode reaction. The latter corresponds to the diffusion process of the reactant or the results of the electrode reaction [28]. It can be seen in Figure 5 that the electrochemical impedance spectroscopy of the AuNPs/AB–DHP/GCE has the smallest semicircular diameter, which means that, as compared to the bare GCE (a) and the AB–DHP/GCE (b), the AuNPs/AB–DHP/GCE (c) had the smallest interface resistance. Therefore, it accelerated the electron transfer rate between the HcySH and the electrode more effectively, indicating the sensor had a short response time.

### 3.3. Investigation on the Electrochemical Oxidation Mechanism of HcySH

**Figure 6:**
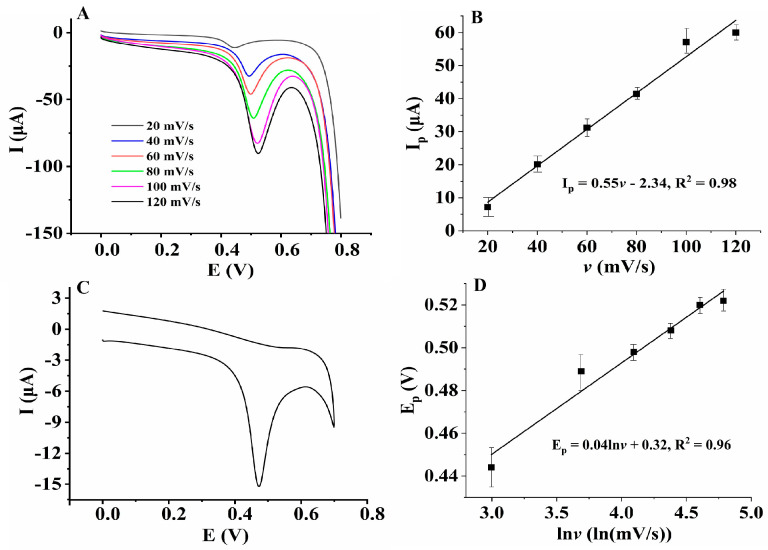
(**A**) Linear sweep voltammograms of 200 μM HcySH on AuNPs/AB–DHP/GCE in 0.15 M PBS (pH = 6.0) with different scan rates. (**B**) Linear relationship between the oxidation peak current of HcySH and scan rate. (**C**) Cyclic voltammogram of 200 μM HcySH on AuNPs/AB–DHP/GCE in 0.15 M PBS (pH = 6.0). (**D**) Linear relationship between the oxidation peak potential of HcySH and the natural logarithm of scan rate.

Linear sweep voltammetry (LSV) and cyclic voltammetry (CV) are the most widely used voltametric techniques for studying the redox reactions of organic and inorganic species because they provide comprehensive information for each step of electrochemical processes with only a modest expenditure of time and effort for the acquisition and interpretation of the data. Therefore, LSV and CV were used to investigate the electrochemical reaction and kinetic process of HcySH on the electrode surface, and the result is shown in Figure 6. Figure 6A shows the electrochemical behavior of 200 µM HcySH on the AuNPs/AB–DHP/GCE at different scan rates. When the scan rate increased from 20 mV/s to 120 mV/s, the oxidation peak current of HcySH increased linearly with the scan rate (Figure 6B), indicating that the oxidation of HcySH on the AuNPs/AB–DHP/GCE was an adsorption-controlled process. In addition, the cyclic voltammogram showed that the electrochemical oxidation of HcySH on the AuNPs/AB–DHP/GCE was also an irreversible process (Figure 6C). According to Laviron’s theory [29], in an electrochemical reaction that is controlled by adsorption and completely irreversible, the relationship between the peak potential and the natural logarithm of the scan rate is described in Equation (1):

(1) $$E_{p}{=E}^{0^{\prime }}+\frac{\mathrm{RT}}{\alpha \mathrm{nF}}\ln\frac{{\mathrm{RTk}}^{0}}{\alpha \mathrm{nF}}+\frac{\mathrm{RT}}{\alpha \mathrm{nF}}\mathrm{lnv}$$

where E^0^′ refers to the formal potential, α refers to the electron transfer coefficient, k^0^ refers to the standard rate constant, n refers to the number of transfer electron, and F refers to the Faraday constant. In this system, the relationship between the peak potential and the natural logarithm of scan rate is: E_p_ (V) = 0.32 + 0.04 ln*v* (mV/s), R^2^ = 0.96 (Figure 6D). According to Equation (1), it can be calculated that the number of the electrons transferred is n = 1.3 ≈ 1. Therefore, the electrochemical oxidation reaction of HcySH is adsorption-controlled, and about one electron is transferred during the process.

**Figure 7:**
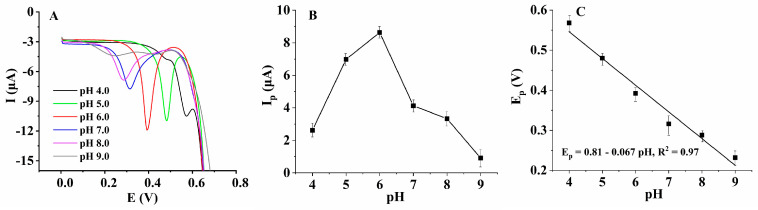
(**A**) Differential pulse voltammograms of 200 μM HcySH on AuNPs/AB–DHP/GCE in 0.15 M PBS with different pH values. (**B**) Linear relationship between the oxidation peak current of HcySH and the pH value. (**C**) Linear relationship between the oxidation peak potential of HcySH and the pH value.

It was necessary to optimize the pH value of the supporting electrolyte because it is not only influenced by the dissociation of the analyte in solution, but it also affected the charge state of the composite film on the electrode surface. The electrochemical behavior of HcySH on the AuNPs/AB–DHP/GCE in the 0.15 M PBS with different pH values was studied using differential pulse voltammetry (DPV) (Figure 7A). The major advantage of DPV is its low capacitive current, which leads to high sensitivity. The results showed that when the pH value of the supporting electrolyte was 6.0, the electrochemical response of HcySH was better (Figure 7B). Furthermore, with the increase in the pH value of the supporting electrolyte, the oxidation peak potential (E_p_) of HcySH decreased linearly: E_p_ = 0.81 − 0.067 pH (R^2^ = 0.97) (Figure 7C). According to our references [30], the slope of the line is equal to −0.059 (m/n) pH, in which m is the number of transferred protons and n is the number of transferred electrons. As the approximated value of n was 1, as described above, the number of transferred protons was also deduced to be approximately equal to 1. Therefore, the reaction of HcySH on the sensing interface was an electrochemical process involving about one electron and about one proton. Combined with the reported literature [31], it was speculated that the electrochemical reaction mechanism of HcySH on the sensor was as follows:

Step 1: HcySH is combined with AuNPs: AuNPs + HcySH→HcyS-AuNPs + H^+^ + e^−^;

Step 2: In an acidic solution: HcyS-AuNPs + H^+^ →(HcyS-AuNPs)H^+^;

Step 3: Scanned for an increased negative potential: (HcyS-AuNPs)H^+^ + e^−^→ HcySH + AuNPs.

### 3.4. Investigation of the Electrocatalytic Mechanism of AuNPs/AB–DHP Nanocomposite Film

#### 3.4.1. Specific Surface Area Increase of the Nanocomposite Film Electrode

**Figure 8:**
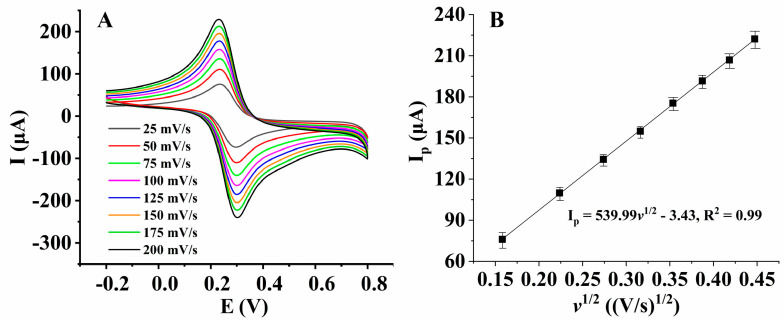
(**A**) Cyclic voltammograms of AuNPs/AB–DHP/GCE in 5 mM Fe(CN)_6_^3−/4−^ + 1 M KCl solution at different scan rates. (**B**) The linear relationship between the reduction peak current of Fe(CN)_6_^3−/4−^ and the square root of the scan rate.

With Fe(CN)_6_^3−/4−^ as the probe, the effective area of the nanocomposite film-modified electrode was measured by cyclic voltammetry (Figure 8A). Figure 8B shows that the peak currents (I_p_) had a good linear relationship with *v*^1/2^ at a temperature of 25 °C. According to the Randles–Sevcik [32] equation (Equation (2)), the values of the effective surface area (A, cm^2^) were calculated.

(2) $$I_{p}=(2{.69\;\times \;10}^{5}{)\;\times \;n}^{3/2}v^{1/2}{\;D}^{1/2}\mathrm{Ac}$$

where D refers to the diffusion coefficient of the probe Fe(CN)_6_^3−/4−^, which is equal to 7.63 × 10^−6^ cm^2^/s, as reported by [33]; c refers to the concentration of the probe Fe(CN)_6_^3−/4−^ (mol/cm^3^); and *v* refers to the scan rate (V/s). As shown in Figure 8B, I_p_ has a linear relationship with *v*^1/2^ as: I_p_ = 539.99 *v*^1/2^ − 3.43, R^2^ = 0.99. For Fe(CN)_6_^3−/4−^, as n = 1, D = 7.63 × 10^−6^ cm^2^/s, c = 5.0 × 10^−3^ mol/cm^3^, the effective surface area of the AuNPs/AB–DHP/GCE was calculated to be 0.145 cm^2^, which was about twice the area of the bare GCE. The larger effective surface area of the AuNPs/AB–DHP/GCE is helpful for the high sensitivity of the HcySH biomedical sensor. Furthermore, supposing that the AuNPs/AB–DHP nanocomposite film was cylindrical on the surface of the GCE, as the surface area of the AuNPs/AB–DHP/GCE was 0.145 cm^2^ and the surface area of the bare GCE was 0.071 cm^2^, the thickness of AuNPs/AB–DHP nanocomposite film was calculated to be 31.85 µm ≈ 32 µm.

#### 3.4.2. Better Enrichment Ability of the Nanocomposite Film Electrode

**Figure 9:**
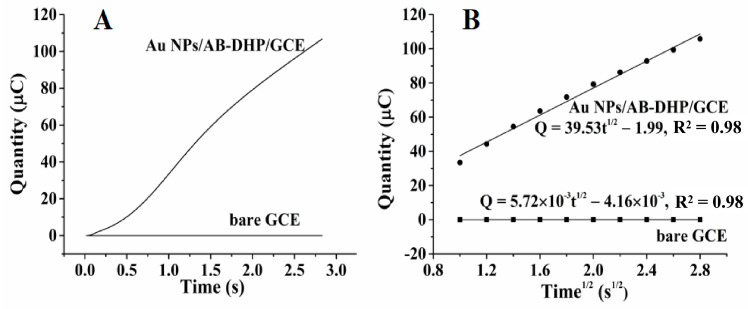
(**A**) Chronocoulomagrams of bare GCE and AuNPs/AB–DHP/GCE in 0.15 M PBS (pH = 6.0) containing 200 μM HcySH. (**B**) The linear relationship between Q and t^1/2^ for bare GCE and AuNPs/AB–DHP/GCE.

Chronocoulometry has been used to verify the enrichment ability of different electrodes, and Figure 9A shows the results. According to Cottrell’s theory [34], the relationship between Q and t^1/2^ is described in Equation (3):

(3) $${\;Q=2\mathrm{ncFAD}}_{0}^{1/2}t^{1/2}\pi^{-1/2}{+Q}_{\mathrm{dl}}{+\mathrm{nFA}\Gamma }_{0}$$

In Equation (3), Q is the quantity of electricity required for the electrochemical reaction of the target molecule on the electrode surface, n refers to the number of transferred electrons, c is the concentration of the target, F is the Faraday constant (96,500 C/mol), A is the electrode area (cm^2^), D is the diffusion coefficient (cm^2^/s) of the target, Q_dl_ is the capacitive quantity of electricity (C), ${\mathrm{nFA}\Gamma }_{0}$ is the Faraday component (C), and $\Gamma_{0}$ is the surface coverage. Combining the data analysis results shown in Figure 9B with Equation (3), the amount of HcySH on the AuNPs/AB–DHP/GCE was calculated to be almost 478 times that on the bare GCE, which ensured the high sensitivity of the sensor.

### 3.5. Investigation of Sensor Performances

#### 3.5.1. Linear Range, Sensitivity and Detection Limit

**Figure 10:**
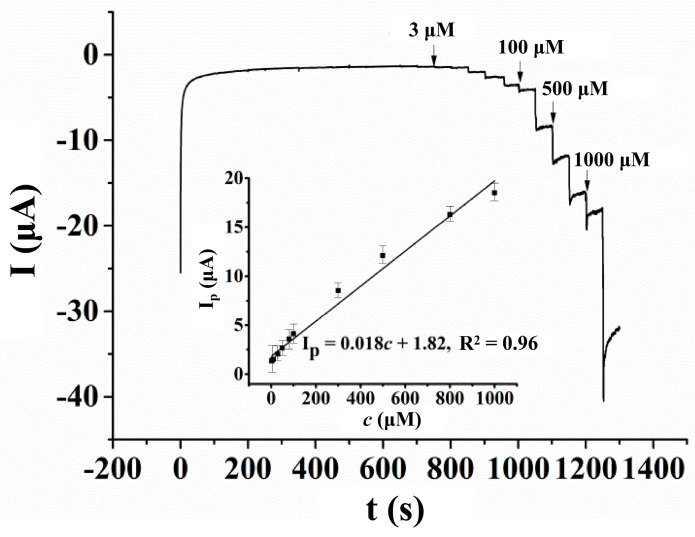
Amperometric response of different concentrations of HcySH on AuNPs/AB–DHP/GCE in 0.15 M PBS (pH = 6.0) with an operation potential of 0.6 V, and the linear relationship between the oxidation peak current of HcySH at different concentrations (inset of Figure 10).

Amperometric measurements were carried out to investigate the linear range of the sensor, and the result is shown in Figure 10. From 200 s to 1250 s, different volumes of the HcySH standard solution were injected into the supporting electrolyte every 50 s. However, from 200 s to 650 s, no obvious electrochemical response was observed due to the low concentration of HcySH in the supporting electrolyte. From 700 s to 1200 s, after the injection of the HcySH standard solution, the concentrations of HcySH in the supporting electrolyte were 3 μM, 6 μM, 10 μM, 30 μM, 50 μM, 70 μM, 100 μM, 300 μM, 500 μM, 700 μM and 1000 μM. The inset of Figure 10 shows the good linear relationship of the oxidation peak current of HcySH on the AuNPs/AB–DHP/GCE, and its concentrations were in the range of 3 μM−1000 μM. The linear equation is: I_p_(μA) = 0.018 c(μM) + 1.82, R^2^ = 0.96. According to the slope of the linear equation, the sensitivity of the sensor was 18 nA/μM. Assuming that the signal-to-noise ratio was equal to 3, as is the standard, the detection limit of the sensor was measured at 0.6 μM, and the response time was 3 s.

The performance of this sensor was compared to other reported HcySH electrochemical sensors, and the results are shown in Table 1. Table 1 indicates that the HcySH electrochemical sensor developed in this paper had a lower oxidation peak potential, wider linear range and lower detection limit than most other reported sensors. Moreover, the fabrication process of the proposed sensor was simple, and the sensitive materials were low cost, which shows its obvious advantages over other systems.

#### 3.5.2. Anti-Interference Ability, Stability and Reproducibility

The anti-interference ability of the AuNPs/AB–DHP/GCE was also examined using amperometric measurements. A foreign species was not considered an interference if it caused a relative error of less than 5% in the analytical signal of the HcySH. The tolerance ratios for 50 µM HcySH were as follows: 50-fold Na^+^, K^+^, Mg^2+^, Ca^2+^; 0.4-fold dopamine, uric acid; and 0.3-fold ascorbic acid, which showed that the sensor was able to resist interference (Figure S1). The stability and reproducibility of the sensor was examined using differential pulse voltammetry. After placing the sensor in the air for 7 days, we then performed parallel measurements (Figure S2). The electrochemical detection signal was able to maintain 93.6% of the initial signal, which indicated the significant stability of the sensor. Under optimal conditions, the peak current of 200 µM HcySH was measured consistently eight times on one AuNPs/AB–DHP/GCE in 0.15 M PBS solution (pH = 6.0) (Figure S3A), and the relative standard deviation (RSD) was calculated at 3.5%. Carrying out the parallel measurements with eight different AuNPs/AB–DHP/GCEs (Figure S3B), the RSD was 6.4%, confirming the reliability of the AuNPs/AB–DHP/GCE.

#### 3.5.3. Determination of HcySH in Serum Samples

**Figure 11:**
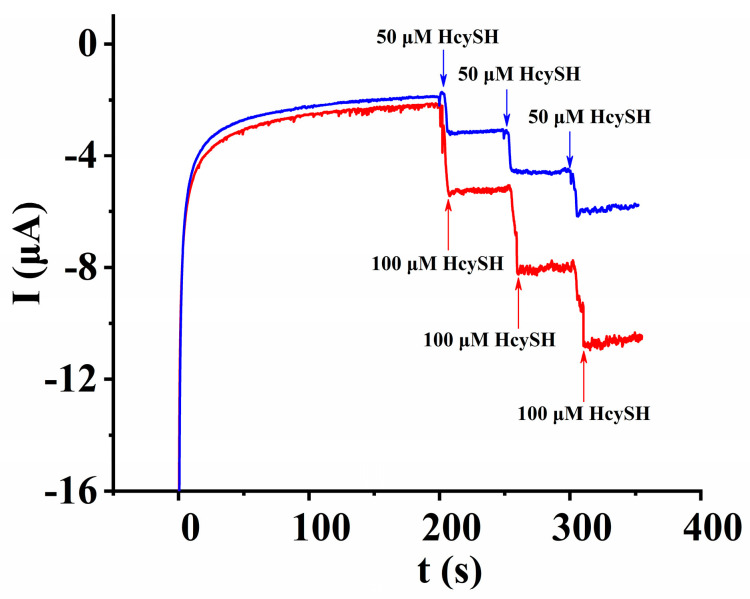
Amperometric response of 50 μM and 100 μM HcySH on AuNPs/AB–DHP/GCE in 0.15 M PBS containing normal human serum sample. The work potential is 0.6 V.

A recovery experiment was carried out to evaluate the practical application of the sensor. While stirred, 300 μL of a normal human serum sample for scientific research was added into 2.7 mL of 0.15 M PBS solution (pH = 6.0), and then a certain amount of HcySH was added into the solution (Figure 11). Six parallel determinations were performed for every sample (n = 6), and the recovery was calculated. As shown in Table S1, the average recovery was 98.3%, which demonstrated the accuracy of the proposed sensor for detecting the concentration of HcySH in serum samples and, thus, its excellent potential in biomedical applications.

## 4. Conclusions

In this work, a novel HcySH nanosensing interface was constructed based on a AuNPs/AB–DHP/GCE that was prepared by a simple and controllable electrochemical deposition and a drop-coating method. The electrochemical experiment results showed that the AuNPs/AB–DHP nanocomposite film had a significant catalytic role in the electrochemical oxidation of HcySH. The surface morphology and conductivity of the nanocomposite was characterized using SEM and EIS. The electrochemical reaction mechanism of HcySH on the sensing interface and the electrocatalytic mechanism of the nanocomposite film were investigated by applying electrochemical techniques. Furthermore, the performance of the constructed HcySH biomedical sensor was evaluated, and the results showed that it had a wide linear range, high sensitivity, a short response time, good resistance to interference, enhanced stability, and reliable accuracy. The sensor can be used for the determination of HcySH in human serum samples and has good application potential.

## Figures and Tables

**Table 1:** Comparison of the performances of different electrochemical sensor for HcySH.

Electrode	Solution	Peak Potential (V)	Method	Linear Range (μM)	Sensitivity (nA/μM)	Detection Limit (nM)	Reference
MWCNTs/GCE	0.2 M PB (pH 7.5)	0.06	LSV	2.5−10 10−100 100−1000	16 8 1	890	[10]
rGO−TiO_2_/GCE	0.1 M PBS (pH 8.0)	0.35	I-t	0.1−80	91.14	24	[13]
AuNPs/rGO/GCE	0.1 M PBS (pH 7.0)	0.12	I-t	2000−14,000	14.8	6900	[27]
Aptamer/AuNPs/GS	0.1 M PBS (pH 7.4)	−0.14	DPV	1−100	630	1000	[35]
DA−GO/SPE	0.1 M PBS (pH 7.0)	0.22	DPV	0.5−50 50−900	381 101	150	[36]
Aptamer/nano−Au/GCE	0.1 M PBS (pH 7.0)	0.8	DPV	0.05−20.0	36	9	[37]
Aptamer/G/GCE	0.25 M PBS (pH 7.0)	0.9	DPV	0.05−20.0	/	5	[38]
Aptamer/Au electrode	0.01 M PBS (pH 7.4)	1.07	DPV	0.2−10	/	10	[39]
ISP/MWCNTPE	Universal buffer (pH 3.5)	0.64	LSV	5−800	/	3300	[40]
BFCNPE	0.1 M PBS (pH 7.0)	0.67	SWV	0.1−10 10.0−80	141 54	50	[41]
Pre-treated PGE	0.05 M PBS (pH 7.4)	0.564	DPV	2−20	20.03	1210	[42]
MWCNTs/GCE	0.15 M PBS (pH 7.0)	−0.2	SWV	0−10	200	660	[43]
MWCNTPE	Universal buffer (pH 5.0) + 0.1 M KCl	0.53	DPV	3−600	8	2080	[44]
MWCNTPE	0.04 M universal buffer (pH 4.0) + 400 μM CHP	0.717	SWV	0.1−210	68	80	[45]
AuNPs/AB–DHP/GCE	0.15 M PBS (pH 6.0)	0.4	I-t	3−1000	18	600	This work

MWCNTs: multiwall carbon nanotubes; GCE: glassy carbon electrode; rGO: reduced graphene oxide; AuNPs: Au nanoparticles; GS: graphene sponge; DA-GO: dopamine-functionalized graphene oxide; SPE: screen-printed electrode; G: graphene; ISP: isoprenaline hydrochloride; MWCNTPE: multiwall carbon nanotubes paste electrode; BFCNPE: benzoylferrocene-modified single-walled carbon nanotube paste electrode; PGE: pencil graphite electrode; CHP: chlorpromazine; AB: acetylene black; DHP: dihexadecyl phosphate.

## Data Availability

Not applicable.

## Supplementary Materials

The following supporting information can be downloaded at: https://www.mdpi.com/article/10.3390/mi14010198/s1, Figure S1: Amperometric response of 50 µM HcySH and other possible coexisting interferents on AuNPs/AB–DHP/GCE in 0.15 M PBS (pH = 6.0) with the operation potential of 0.6 V, Figure S2: Differential pulse voltammograms of 200 μM HcySH on AuNPs/AB–DHP/GCE in 0.15 M PBS (pH = 6.0) at different time, Figure S3: Differential pulse voltammograms of 200 μM HcySH on AuNPs/AB–DHP/GCE in 0.15 M PBS (pH = 6.0). (A) Parallel detection with one AuNPs/AB–DHP/GCE for eight times. (B) Parallel detection with eight different AuNPs/AB–DHP/GCEs, Table S1: Results of the recovery experiment.
