# Supplementary material for: An Amperometric Biomedical Sensor for the Determination of Homocysteine Using Gold Nanoparticles and Acetylene Black-Dihexadecyl Phosphate-Modified Glassy Carbon Electrode
Source: Micromachines (Basel). 2023 Jan 13;14(1):198. doi: 10.3390/mi14010198 (PMC9865262; doi:10.3390/mi14010198)
Supplement: Supplementary file 1 [file micromachines-14-00198-s001.zip › micromachines-2081650-supplementary.pdf]

Supporting Information

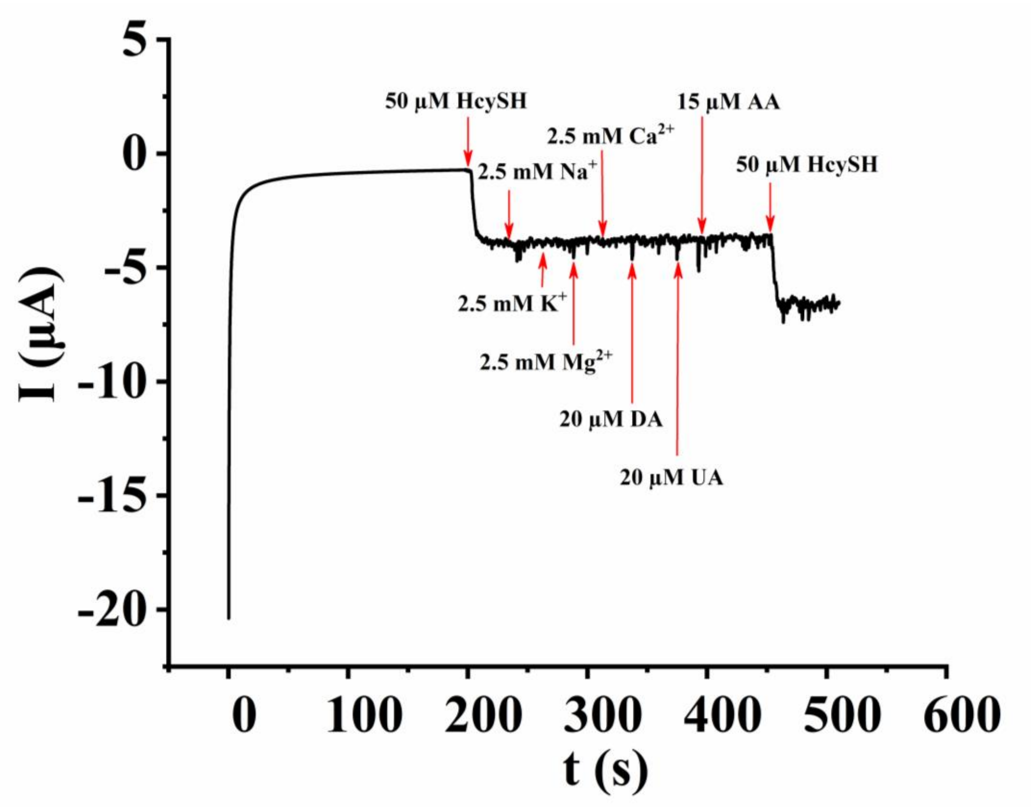

Figure S1. Amperometric response of 50 µM HcySH and other possible coexisting interferents on AuNPs/AB−DHP/GCE in 0.15 M PBS (pH = 6.0) with the operation potential of 0.6 V.

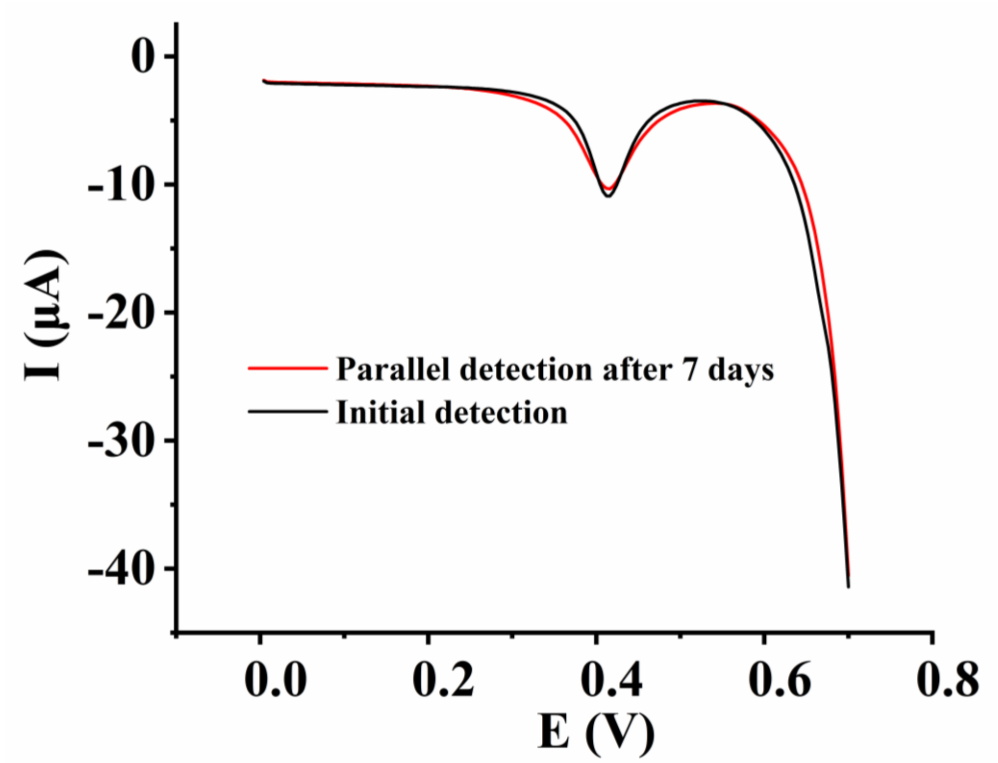

Figure S2. Differential pulse voltammograms of 200 μM HcySH on AuNPs/AB−DHP/GCE in 0.15 M PBS (pH = 6.0) at different time.

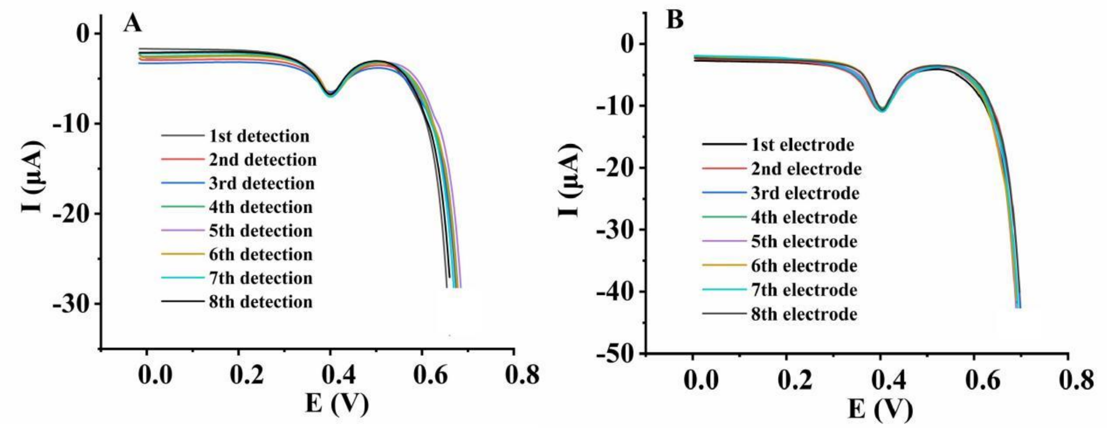

Figure S3. Differential pulse voltammograms of 200 μM HcySH on AuNPs/AB−DHP/GCE in 0.15 M PBS (pH = 6.0). (A) Parallel detection with one AuNPs/AB−DHP/GCE for eight times. (B) Parallel detection with eight different AuNPs/AB−DHP/GCEs.

Table S1. Results of the recovery experiment.

Human blood serum	Added (μM)	Found (μM)	Recovery (%)
Sample 1	50	47.3	94.5
Sample 2	50	51.9	103.9
Sample 3	50	46.7	93.4
Sample 4	100	92.9	92.9
Sample 5	100	107.6	107.6
Sample 6	100	97.3	97.3
